# Risk score to predict fibrosis among Mexican adults: results of the Health Workers Cohort Study

**DOI:** 10.3389/fmed.2026.1699322

**Published:** 2026-03-10

**Authors:** Edgar Denova-Gutiérrez, Berenice Rivera-Paredez, Amado D. Quezada-Sánchez, Norberto Chávez-Tapia, Paloma Muñoz-Aguirre, Brianda Armenta-Guirado, Daniela Contreras, Yvonne N. Flores, Rafael Velázquez-Cruz, Jorge Salmerón

**Affiliations:** 1 Departamento de Estudios Experimentales Rurales, Instituto Nacional de Ciencias Médicas y Nutritición Salvador Zubirán, Mexico City, Mexico; 2 Center of Policy, Population and Health Research, Faculty of Medicine, National Autonomous University of Mexico, Mexico City, Mexico; 3 Center for Evaluation and Surveys Research, National Institute of Public Health, Cuernavaca, Mexico; 4 Obesity and Digestive Disease Unit, Medica Sur Clinic and Foundation, Mexico City, Mexico; 5 National Council of Humanities, Sciences, and Technologies (CONAHCYT), Mexico City, Mexico; 6 Center for Population Health Research, National Institute of Public Health, Cuernavaca, Mexico; 7 Department of Health Sciences, University of Sonora, Hermosillo, Mexico; 8 School of Medicine and Health Science, Tecnológico de Monterrey, Santiago de Querétaro, Mexico; 9 Unit of Epidemiological and Health Services Research, Mexican Social Security Institute, Cuernavaca, Mexico; 10 UCLA Department of Health Policy and Management, Center for Cancer Prevention and Control Research, UCLA Kaiser Permanente Center for Health Equity, Fielding School of Public Health and Jonsson Comprehensive Cancer Center, Los Angeles, CA, United States; 11 Genomics of Bone Metabolism Laboratory, National Institute of Genomic Medicine, Mexico City, Mexico

**Keywords:** adult population, Fibroscan, fibrosis, METAVIR, receiving operating characteristic (ROC)

## Abstract

**Background:**

Fibrosis, a severe complication of metabolic-associated fatty liver disease, represents a major concern in the disease progression. Although liver biopsy remains the gold standard for fibrosis diagnosis, its invasive nature and high cost highlight the need for reliable non-invasive alternatives.

**Aim:**

This study aimed to develop a simple, non-invasive fibrosis risk score based on clinical and biochemical variables in a Mexican population.

**Methods:**

A total of 295 participants from the Health Workers Cohort Study were included. Fibrosis was assessed using transient elastography (FibroScan), which served as the reference standard for the development and validation of the risk score. Participants were classified according to the Meta-analysis of Histological Data in Viral Hepatitis (METAVIR) system. The diagnostic performance of candidate predictors was evaluated using receiver operating characteristic (ROC) curve analysis, and optimal cutoffs for fibrosis detection were identified.

**Results:**

The fibrosis risk score developed for the Mexican population, including sex, triglycerides, glucose, waist circumference, hypertension, high-density lipoprotein cholesterol, body mass index (BMI), aspartate aminotransferase (AST), alanine aminotransferase (ALT), gamma-glutamyl transferase (GGT), and insulin, achieved an area under the ROC (AUROC) of 81.1% (95% CI, 74.4, 87.8). In the subsample with available insulin measurements, the AUROC was comparable (79.8, 95% CI, 72.9–86.8). When compared with existing fibrosis scores, including the BARD score (based on BMI, aspartate aminotransferase/alanine aminotransferase (AST/ALT) ratio, and type 2 diabetes) and the AST/ALT ratio, the newly developed score demonstrated superior diagnostic accuracy for fibrosis detection.

**Conclusion:**

This fibrosis risk score, based on routinely available clinical and biochemical data, demonstrated high diagnostic accuracy in the Mexican adult population. As a non-invasive tool, it may facilitate the early identification of fibrosis in primary care settings and reduce the need for liver biopsy. Further validation in larger and more diverse populations is warranted.

## Introduction

Globally, the rising prevalence of obesity, type 2 diabetes (T2D), and other metabolic disorders has positioned metabolic-associated fatty liver disease (MAFLD) as the leading cause of chronic liver disease (1, 2). MAFLD encompasses a wide spectrum of liver injury, ranging from hepatic fat accumulation (steatosis) to non-alcoholic steatohepatitis (NASH). Individuals with NASH are at a substantially higher risk of progression to fibrosis, cirrhosis, and related complications (2).

Non-alcoholic fatty liver disease (NAFLD) is the most prevalent chronic liver condition worldwide, affecting an estimated 17–46% of the adult population (3). In Mexico, available data suggest that more than 60% of adults are affected by NAFLD (4), with approximately 52% presenting NASH and 47% meeting the diagnostic criteria for MAFLD (5). As NASH progresses, liver injury worsens, leading to the development of fibrosis, underscoring the importance of accurately assessing the fibrosis stage in affected individuals.

Although liver biopsy remains the gold standard for fibrosis staging, its clinical use is constrained by several limitations, such as its invasive nature, patient discomfort, high cost, sampling variability, and dependence on operator expertise (6, 7). Non-invasive imaging modalities—such as transient elastography (FibroScan) and magnetic resonance elastography—offer high diagnostic accuracy but remain costly and are not universally accessible (8, 9). As a result, the development of non-invasive approaches for fibrosis detection has become a clinical priority (10).

Several clinical and biochemical factors have been consistently associated with liver fibrosis, including older age, T2D, metabolic syndrome, elevated body mass index (BMI), an increased aspartate aminotransferase (AST) to alanine aminotransferase (ALT) ratio (AAR), and reduced platelet count. These variables form the basis of multiple non-invasive fibrosis assessment tools. While some rely on simple calculations (e.g., AAR), others—such as the NAFLD fibrosis score, FIB-4, and the AST-to-platelet ratio index (APRI)—incorporate more complex algorithms (11).

Although these scoring systems have been validated against liver biopsy and imaging-based techniques, their diagnostic performance varies across populations and clinical settings (12–15). This variability highlights the need for population-specific tools, particularly in settings where access to liver biopsy or advanced imaging is limited and where the prevalence of NASH is high (5), such as in Mexico.

Therefore, the objective of this study was to develop a simple, non-invasive scoring system for the detection of liver fibrosis using routinely available clinical and biochemical variables.

## Materials and methods

### Study population

This study is based on data from the Health Workers Cohort Study (HWCS), a longitudinal investigation initiated between 2004 and 2006. The primary objective of the HWCS is to generate evidence to inform the development of effective programs and policies aimed at reducing the burden of chronic diseases in the Mexican population. The study design, protocols, and procedures have been described in detail elsewhere (16).

Participants, who were predominantly female, were primarily employees of the Mexican Social Security Institute (IMSS, Spanish acronym) and their relatives residing in Cuernavaca, Morelos. For the present analysis, the inclusion criteria were as follows: adults aged >20 years, availability of alanine aminotransferase (ALT), aspartate aminotransferase (AST), and gamma-glutamyl transferase (GGT) measurements, a valid FibroScan assessment, and complete data on relevant covariates. Participants without FibroScan measurements were excluded. Based on these criteria, a total of 300 participants, with data collected between 2017 and 2019, were included in the analysis.

This study was conducted in accordance with the principles of the Declaration of Helsinki. All participants provided written informed consent prior to enrollment and data collection, and the IMSS Institutional Review Board approved all procedures. The study protocol was reviewed and approved by the Institutional Review Boards of all participating institutions, including the IMSS (12CEI 09006 14) and the National Institute of Public Health (13CEI 17 007 36).

### Elastography assessment

Liver stiffness measurements were performed using transient elastography (FibroScan® FS402, probe M; Echosens, Paris, France), which served as the reference standard for fibrosis classification in this study. Examinations were conducted on the right lobe of the liver, with participants in the dorsal decubitus position and the right arm in maximal abduction. Liver tissue with a minimum thickness of 6 cm, free of large vascular structures, was identified using A-mode sonography, as recommended to ensure reliable measurements (17–19).

A minimum of 10 valid measurements was obtained according to the manufacturer’s guidelines. At least six valid measurements were required for inclusion, ensuring that the interquartile range did not exceed 30% of the median liver stiffness value. Fibrosis stages were classified according to the Meta-analysis of Histological Data in Viral Hepatitis (METAVIR) scoring system as follows: F0 (no fibrosis), F1 (portal fibrosis without septa), F2 (portal fibrosis with few septa), F3 (numerous septa without cirrhosis), and F4 (cirrhosis) (20, 21). For this analysis, fibrosis status was defined dichotomously: F0 indicated no fibrosis, while F1–F4 indicated the presence of fibrosis.

Based on prior studies evaluating the diagnostic performance of transient elastography for hepatic fibrosis (9, 22, 23), liver stiffness cut-off values were defined as follows: 7.0–7.9 kPa for F1, 8.0–9.5 kPa for F2, 9.5–13.9 kPa for F3, and ≥14.0 kPa for F4.

### Biomarkers and covariate assessment

After an overnight fast (>8 h), a 20-mL venous blood sample was collected from each participant, and all biochemical assays were processed using a Selectra XL analyzer (Randox, Northern, Irish), in accordance with the guidelines of the International Federation of Clinical Chemistry and Laboratory Medicine.

Serum ALT, AST, and GGT levels were measured using enzymatic assays based on catalytic activity, monitored via the decrease in nicotinamide adenine dinucleotide (NADH) absorbance at 340 nm through a lactate dehydrogenase-coupled reaction. Serum glucose concentrations were measured using the glucose oxidase method. Serum insulin levels were determined by a solid-phase direct radioimmunoassay (Coat-A-Count®, Diagnostic Products, Los Angeles, CA, USA). triglycerides (TG) were measured using a colorimetric enzymatic method after lipase-mediated hydrolysis, and high-density lipoprotein cholesterol (HDL-c) was assessed using the clearance method.

Body mass index (BMI) was calculated as weight in kilograms (kg) divided by height in meters squared (m^2^). Waist circumference (WC) was measured to the nearest 0.5 cm at the midpoint between the lower margin of the rib cage and the iliac crest.

Blood pressure was measured twice by trained nursing staff using an automated sphygmomanometer (OMRON HEM-907, Kyoto, Japan). The first measurement was obtained after a 5-min rest period with participants seated and the dominant arm supported at the heart level; the second measurement was taken after an additional 5-min rest interval.

Participants also completed standardized self-administered questionnaires collecting information on demographic characteristics (age and sex), health status (including prior diagnosis of type 2 diabetes), and lifestyle factors, such as smoking and alcohol consumption (16).

### Statistical analysis

This study was designed as a derivation analysis to develop a simple, non-invasive fibrosis risk score for Mexican adults based on routinely available clinical and biochemical variables. All modeling strategies were selected to prioritize discrimination, interpretability, and potential applicability in primary care settings. Fibrosis status defined by transient elastography served as the reference standard outcome for model development and validation.

Continuous variables are presented as means and standard deviations (SD), while categorical variables are summarized as frequencies and percentages.

We first evaluated the discriminatory performance of individual predictors—including insulin, BMI, WC, glucose, TG, the AST/ALT ratio (17), and the BARD index (24–26)—using the area under the receiver operating characteristic curve (AUROC). We then assessed classification performance for selected pairs of predictors (insulin and WC, glucose and WC, and TG and WC). For each pair, AUROC values were calculated using both the average of standardized predictors and a linear predictor derived from multivariable probit regression models. Average AUROC estimates were obtained across five-fold cross-validation samples. Subsequently, classification performance was evaluated using a linear predictor derived from a multivariable Bayesian probit regression model. In this approach, the fibrosis risk score was computed by weighting each predictor by the posterior mean of its corresponding probit coefficient.

The model included indicator variables for TG ≥ 150 mg/dL, glucose ≥100 mg/dL, systolic blood pressure (SBP) ≥ 135 mmHg or diastolic blood pressure (DBP) ≥ 85 mmHg, and HDL-c < 40 mg/dL in male or <50 mg/dL in female participants; abdominal obesity was assessed by WC; and additional variables included sex, AST/ALT ratio, AST, GGT, and insulin (with AST, GGT, and insulin entered as standardized variables). This specification is hereafter referred to as the “saturated” model. Five-fold cross-validation was again performed to assess the model’s out-of-sample classification accuracy.

Because insulin data were missing for 71 participants, all analyses were repeated in the subsample without insulin measurements. Diffuse half-normal priors with location parameters equal to zero were specified for all metabolic predictors, reflecting their assumed positive association with fibrosis risk. Detailed model specifications and estimation procedures are provided in Appendix 1.

Using the full analytic sample (*n* = 295), optimal cut-off points for the fibrosis risk score were determined using established optimization procedures (27). Cut-offs were optimized across three assumed fibrosis prevalence levels (15, 30, and 50%) and three misclassification cost ratios (false-negative to false-positive costs of 1, 2, and 3). The optimized cut-off, assuming a fibrosis prevalence of 50% and equal misclassification costs, corresponds to the Youden Index.

All statistical analyses were performed using Stata version 18.5 (StataCorp LLC. Texas, USA). Bayesian analyses were conducted using JAGS version 4.3.1 through the rjags package in R, and cutoff optimization was performed using the OptimalCutpoints package in R version 4.4.2 (Free Software Foundation, Inc., Boston, USA).

## Results

### Characteristics of the study population

A total of 300 patients with available FibroScan data and complete sociodemographic, biological, and clinical information were reviewed. Five patients were excluded due to missing clinical, biological, or sociodemographic data, resulting in a final analytic sample of 295 individuals. Of these, 85.4% were classified as free of fibrosis, while 3.3% were classified as F1, 6.0% as F2, 4.0% as F3, and 1.3% as F4 (data not shown). The mean age of the participants was 59.1 years (SD = 12.0), and 74.9% were female. Additionally, 67.8% of participants were classified as overweight or obese, with the prevalence being higher among those with fibrosis than among those without fibrosis (86% vs. 64.7%, respectively). Furthermore, 14.6% of participants had T2D, with a higher prevalence observed among individuals with fibrosis (27.9%) (Table 1).

**Table 1:** Main characteristics of the subjects included in the study according to the fibrosis status.

Variable	Total sample *n* = 295	Without fibrosis *n* = 252	With fibrosis *n* = 43	*p*
	Mean (SD) or *n* (%)			
Age, years	59.1 (12.0)	58.8 (12.1)	60.8 (11.5)	0.331
Sex				
Male	74 (25.1%)	63 (25.0%)	11 (25.6%)	0.935
Female	221 (74.9%)	189 (75.0%)	32 (74.4%)	
BMI, kg/m^2^	28.2 (5.3)	27.6 (4.9)	32.1 (6.2)	<0.001
Normal	95 (32.2%)	89 (35.3%)	6 (14.0%)	<0.001
Overweight	109 (36.9%)	97 (38.5%)	12 (27.9%)	
Obesity	91 (30.8%)	66 (26.2%)	25 (58.1%)	
Waist circumference, cm	95.0 (11.9)	93.4 (10.9)	104.2 (13.5)	<0.001
No abdominal obesity	117 (39.7%)	111 (44.0%)	6 (14.0%)	<0.001
Abdominal obesity	178 (60.3%)	141 (56.0%)	37 (86.0%)	
Visceral fat, kg	19.2 (12.5)	19.0 (12.6)	20.3 (11.5)	0.509
Triglycerides, mg/dL	156.4 (76.1)	152.4 (70.8)	179.4 (99.8)	0.031
Triglycerides <150	160 (54.2%)	139 (55.2%)	21 (48.8%)	0.442
Triglycerides ≥150	135 (45.8%)	113 (44.8%)	22 (51.2%)	
Total cholesterol, mg/dL	196.0 (42.9)	196.7 (40.9)	191.3 (53.2)	0.442
HDL-c, mg/dL	52.2 (14.1)	52.8 (14.2)	48.4 (13.1)	0.056
Not low HDL-c	179 (60.7%)	161 (63.9%)	18 (41.9%)	0.006
Low HDL-c	116 (39.3%)	91 (36.1%)	25 (58.1%)	
LDL-c, mg/dL	114.2 (41.3)	114.6 (39.4)	111.3 (52.1)	0.636
Glucose, mg/dL	109.2 (34.6)	105.3 (27.0)	132.6 (58.1)	<0.001
Glucose <100 mg/dL	148 (50.2%)	138 (54.8%)	10 (23.3%)	<0.001
Glucose ≥100 mg/dL	147 (49.8%)	114 (45.2%)	33 (76.7%)	
Systolic blood pressure, mmHg	122.9 (19.0)	121.6 (19.0)	130.1 (17.1)	0.006
Diastolic blood pressure, mmHg	75.7 (9.9)	75.6 (10.0)	75.8 (9.5)	0.898
Without hypertension	201 (68.1%)	176 (69.8%)	25 (58.1%)	0.128
With hypertension	94 (31.9%)	76 (30.2%)	18 (41.9%)	
Type 2 diabetes [T2D]				
Without T2D	252 (85.4%)	221 (87.7%)	31 (72.1%)	0.007
With T2D	43 (14.6%)	31 (12.3%)	12 (27.9%)	
Insulin, ng/mL	0.4 (0.5)	0.4 (0.4)	0.7 (0.7)	<0.001
ALT, mg/dL	35.2 (23.9)	32.6 (20.1)	50.3 (36.2)	<0.001
AST, mg/dL	31.6 (16.7)	29.3 (13.2)	44.8 (26.3)	<0.001
GGT, U/L	40.1 (36.2)	37.5 (35.1)	55.3 (39.5)	0.003

BMI, body mass index; HDL-c, high-density lipoprotein cholesterol; LDL-c, low-density lipoprotein cholesterol; AST, aspartate aminotransferase; ALT, alanine aminotransferase; GGT, gamma-glutamyl transferase.

### Predictor weights in the Mexican fibrosis risk score

**Figure 1:**
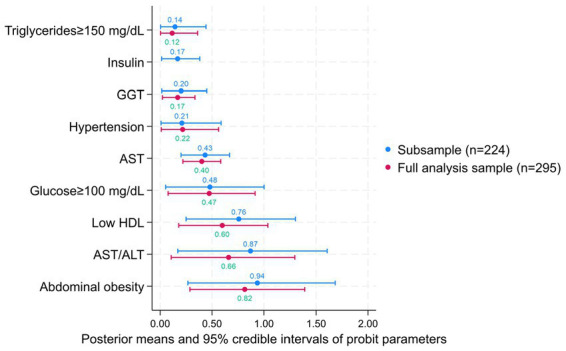
Posterior means of predictor coefficients from a Bayesian probit model for fibrosis and their 95% percentile-based credible intervals. Insulin, GGT, and AST were standardized before inclusion in the model. An indicator variable of whether the participant is female was included along with the other predictors, its coefficient had a posterior mean of −0.68 (95% CI, −1.31, −0.06) in the subsample and a posterior mean of −0.49 (95% CI, −1.02, 0.04) in the total sample. Posterior means (95% CI) for model probit constants were −3.15 (−4.30, −2.08) in the subsample and in the total sample −2.79 (−3.70, −1.98).

The metabolic predictor weights used to calculate our fibrosis risk score are shown in Figure 1. Abdominal obesity, the AST/ALT ratio, and low HDL-c had the highest weights, whereas high triglycerides, insulin, GGT, and hypertension received the lowest weights.

### Performance of the Mexican fibrosis risk score

The diagnostic performance of the newly developed fibrosis risk score and existing non-invasive indices for fibrosis detection is presented in Table 2. In the full analytic sample, the fibrosis risk score achieved an AUROC of 81.1 (95% CI: 74.4–87.8). In the subsample with available insulin measurements, the AUROC was 79.8 (95% CI: 72.9–86.8). Classification accuracy using five-fold cross-validation yielded an AUROC of 79.0 (95% CI: 72.1–85.8) in the full sample and 79.2 (95% CI: 71.2–87.3) in the subsample, indicating stable discriminatory performance.

**Table 2:** Areas under receiver operating characteristic (AUROC) curves by classifier.

Classifier	AUROC (95% CI)	
	Subsample^a^ *n* = 224	Full analysis sample *n* = 295
Insulin	71.1 (62.1, 80.0)	
BMI	75.7 (66.0, 85.3)	72.6 (63.6, 81.6)
Waist circumference	75.5 (66.2, 84.7)	72.9 (64.3, 81.4)
Glucose	70.4 (60.2, 80.5)	70.8 (62.4, 79.3)
Triglycerides	60.2 (50.3, 70.2)	59.1 (50.9, 67.2)
Insulin and waist circumference		
Average	77.8 (69.7, 85.9)	
Model^b^	77.6 (69.1, 86.2)	
Five-fold validation^c^	75.6 (66.3, 85.0)	
Glucose and waist circumference		
Average	78.0 (69.8, 86.3)	75.6 (68.2, 83.1)
Model^b^	77.9 (69.6, 86.3)	75.2 (67.6, 82.8)
Five-fold validation^c^	77.1 (67.9, 86.4)	74.5 (66.8, 82.3)
Triglycerides and waist circumference		
Average	75.3 (66.3, 84.2)	71.2 (63.1, 79.4)
Model^b^	77.1 (68.1, 86.0)	73.7 (65.3, 82.1)
Five-fold validation^c^	75.4 (65.6, 85.2)	72.3 (63.4, 81.1)
Fibrosis risk score^d^		
Model^b^	79.8 (72.9, 86.8)	81.1 (74.4, 87.8)
Five-fold validation^c^	79.2 (71.2, 87.3)	79.0 (72.1, 85.8)
AST/ALT ratio	48.2 (36.8, 59.6)	47.8 (38.5, 57.0)
BARD	66.3 (57.0, 75.6)	68.5 (60.6, 76.5)

AUROC, area under the receiver operating characteristic curve expressed as a percentage.

a Subsample with insulin data.

b Linear predictor of a multivariable probit regression model.

c Average AUROC over validation samples using *k*-fold cross-validation with *k* = 5.

d Linear predictor from a Bayesian probit model with coefficients set at their posterior means. Predictors included indicator variables of triglycerides ≥150, glucose ≥100, systolic blood pressure (SBP) ≥135 or diastolic blood pressure (DBP) ≥85, high-density lipoprotein cholesterol (HDL-c) <40 if male or HDL-c <50 if female, body mass index (BMI) category, sex, aspartate aminotransferase (AST), gamma-glutamyl transferase (GGT), abdominal obesity (waist circumference ≥102 in male and ≥88 in female patients), and insulin (in the subsample).

### Comparison with existing fibrosis scores

**Figure 2:**
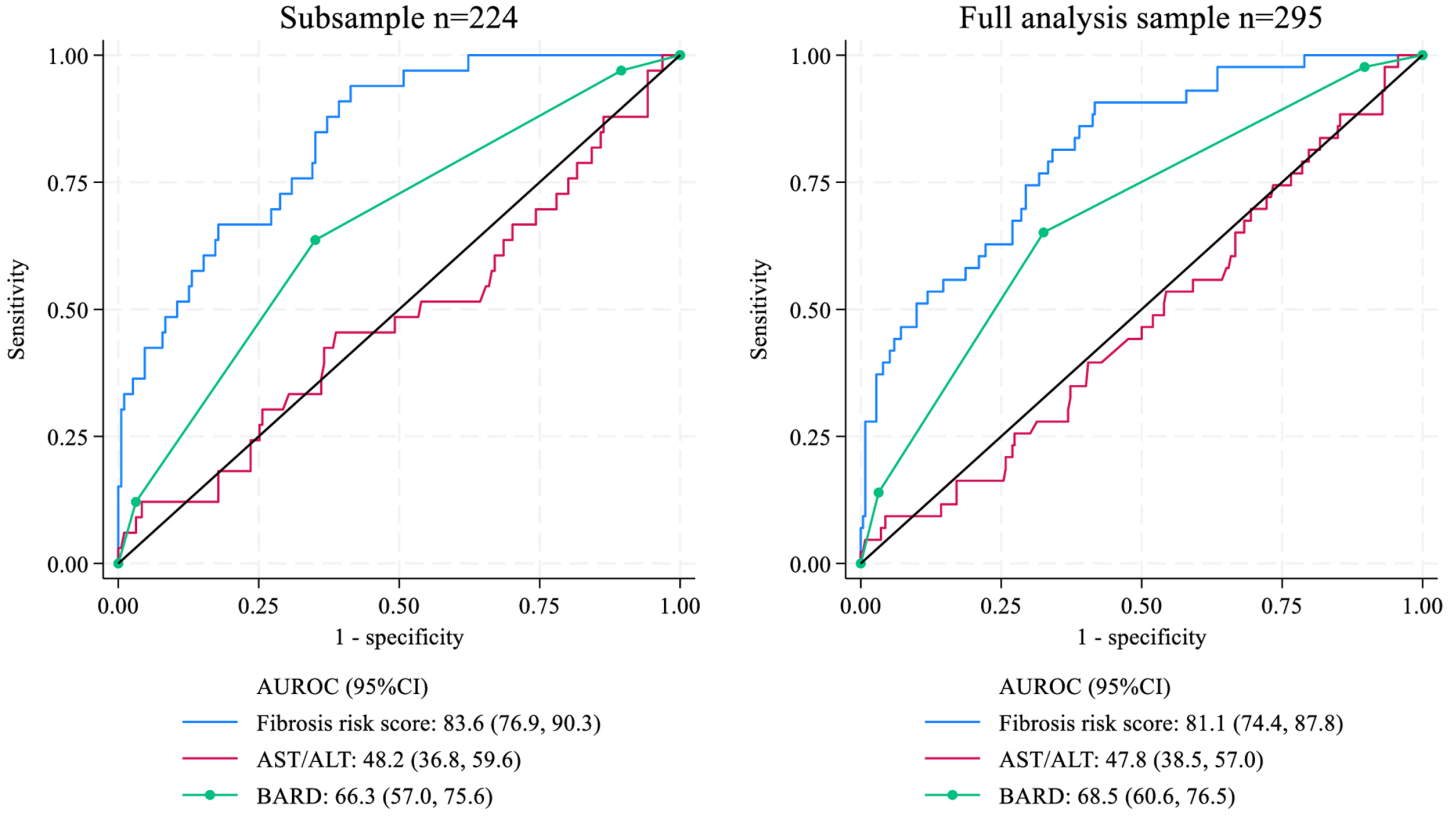
Receiver operating curves (ROC) by classifier and analysis sample.

**Figure 3:**
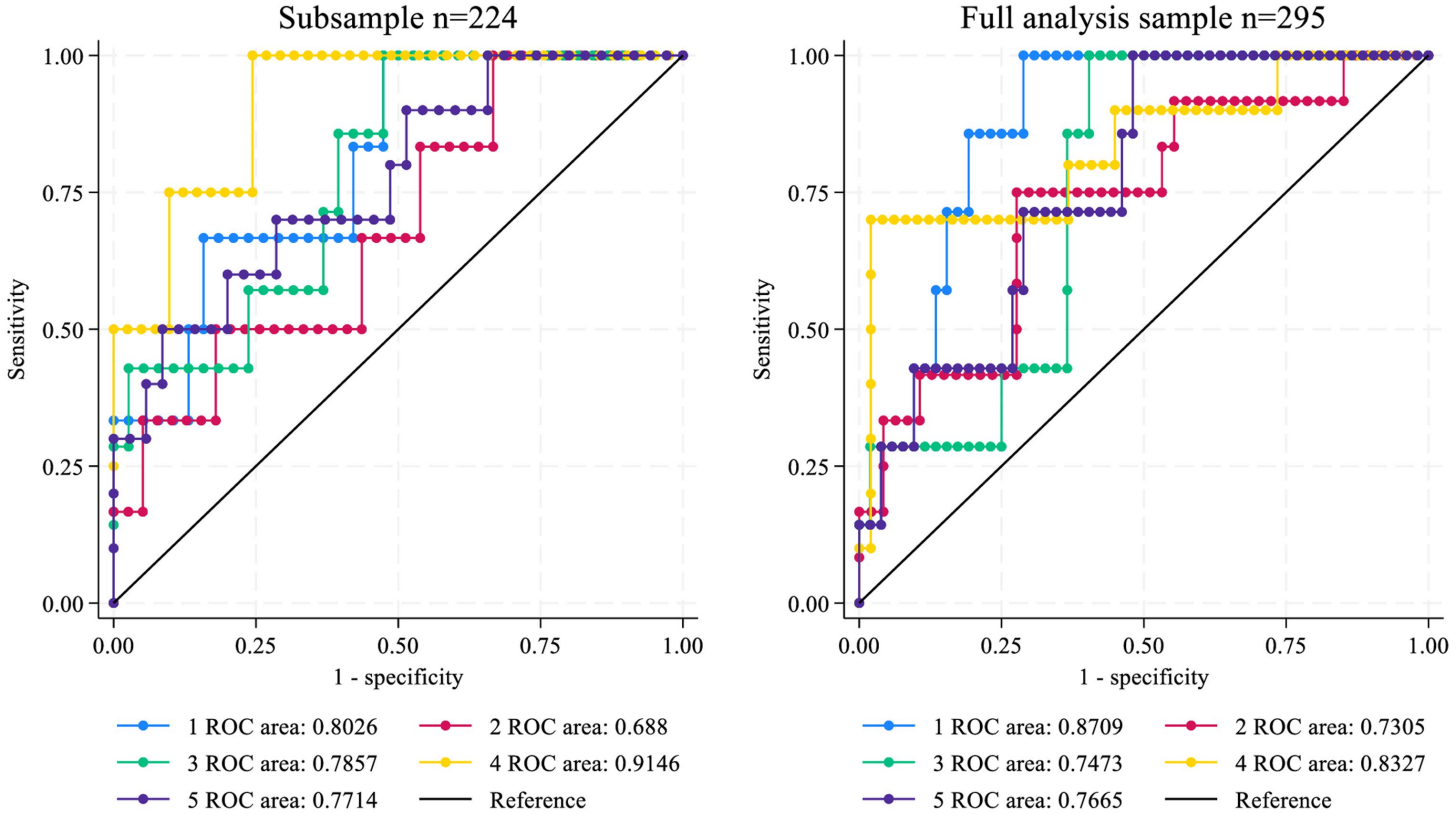
Receiver operating curves (ROC) of the 5-fold validation partitions.

The diagnostic accuracy of the Mexican fibrosis risk score was compared with existing non-invasive fibrosis indices, including the AST/ALT ratio and the BARD score, using AUROC curves (Figure 2). The Mexican fibrosis risk score demonstrated the highest discriminatory performance for fibrosis status (AUROC = 81.1; 95% CI: 74.4–87.8), followed by the BARD score (AUROC = 68.5; 95% CI: 60.6–76.5). The AST/ALT ratio showed no significant discriminatory capacity (AUROC = 47.8; 95% CI: 38.5–57.0) in the full analytic sample (Figure 3).

### Optimization of cut-offs

The results of the cutoff optimization under varying assumptions of fibrosis prevalence and misclassification cost ratios are presented in Table 3. For example, assuming a fibrosis prevalence of 15% and a false-negative cost twice that of a false-positive cost, the optimal cut-off for the fibrosis risk score derived from the saturated model was −0.16. Under these conditions, the model achieved a positive predictive value of 70.3% and a negative predictive value of 89.8%. Specificity was substantially higher than sensitivity (97.2% vs. 37.2%, respectively), reflecting prioritization of minimizing false-positive classifications in low-prevalence settings.

**Table 3:** Optimization of cutoffs under different fibrosis prevalence and misclassification cost ratios.

Miss-classification cost ratio^a^	Fibrosis prevalence	Classification cutoff^c^	Sensitivity	Specificity	Positive predictive value	Negative predictive value
1	15%	0.09	27.9%	99.2%	86.1%	88.6%
	30%	−0.16	37.2%	97.2%	85.2%	78.3%
	50%	−1.36^b^	90.7%	58.3%	68.5%	86.2%
2	15%	−0.16	37.2%	97.2%	70.3%	89.8%
	30%	−1.36	90.7%	58.3%	48.3%	93.6%
	50%	−1.36	90.7%	58.3%	68.5%	86.2%
3	15%	−0.45	46.5%	92.9%	53.5%	90.8%
	30%	−1.36	90.7%	58.3%	48.3%	93.6%
	50%	−1.36	90.7%	58.3%	68.5%	86.2%

a Ratio between false negative costs and false positive costs. A ratio of 2 implies that the false negative cost is twice that of the false positive cost.

b Equivalent to optimizing the classification cutoff with the Youden index criteria.

c The classifier corresponds to the linear predictor obtained from a probit Bayesian model when predictor coefficients are set at their posterior means. Predictors included indicator variables of triglycerides ≥150, glucose ≥100, systolic blood pressure (SBP) ≥135 or diastolic blood pressure (DBP) ≥85, high density lipoprotein cholesterol (HDL-c) < 40 in male or HDL-c <50 in female participants, abdominal obesity (waist circumference ≥102 in men and ≥88 in women), and insulin (in the subsample). Additional predictors included sex, aspartate aminotransferase (AST), the AST/ALT ratio, and gamma-glutamyl transferase (GGT).

## Discussion

The search for non-invasive diagnostic tools for liver fibrosis has gained importance in recent years, particularly in settings characterized by pronounced health disparities and limited access to specialized healthcare resources. Several non-invasive fibrosis scores have been developed to reduce reliance on liver biopsy among populations at risk. However, to date, none of these tools have been specifically developed or systematically evaluated in a Mexican population using exclusively measurements that can be obtained at the primary care level.

In this study, we developed a non-invasive fibrosis risk score based on routinely available clinical, anthropometric, and biochemical variables to predict fibrosis status in Mexican adults. In addition, we evaluated the performance of existing non-invasive indices, such as the BARD and ALT/AST ratio, which have been externally validated in diverse populations, often with heterogeneous results. Our findings indicate that sex, elevated triglycerides and glucose levels, hypertension, low HDL-c, abdominal obesity, AST, AST/ALT ratio, GGT, and insulin (in the subsample) contribute to fibrosis prediction. Abdominal obesity, the AST/ALT ratio, and low HDL-c showed the highest probit coefficients, whereas triglycerides, insulin, GGT, and hypertension exhibited more modest associations. However, when interpreting the size of associations in our model, it should be noted that predictors were not all measured on the same scale. Overall, the Mexican fibrosis risk score demonstrated good discriminatory performance, with AUROC values of 81.1% in the full analytic sample and 79.8% in the subsample with insulin measurements. In contrast, the BARD score showed moderate accuracy (AUROC: 68.5 and 66.3% in the full sample and subsample, respectively), while the AST/ALT ratio alone showed no significant discriminatory capacity (AUROC: ~50%), indicating that this marker by itself was not useful for fibrosis classification in our study population. The results from the saturated probit regression model suggest that this association was suppressed when other predictors were excluded from the analysis.

Consistent with previous evidence, a high proportion of participants with fibrosis in our study were living with overweight or obesity (86%), reinforcing the well-established association between excess adiposity and liver fibrosis. Additionally, T2D and insulin resistance—frequent metabolic complications of obesity—were more prevalent among participants with fibrosis. In our analysis, 28% of individuals with fibrosis had a prior diagnosis of T2D, a finding that aligns with prior studies conducted in different populations (15, 26–29).

Multiple studies have identified key predictors of fibrosis, including sex (29), obesity (28, 30), ALT, AST, the AST/ALT ratio (29), insulin resistance (31, 32), hypertension (32, 33), and hypertriglyceridemia (30). Our results reinforce the relevance of these factors—particularly abdominal obesity, altered glucose metabolism, dyslipidemia (low HDL-c and elevated triglycerides), hypertension, and liver enzyme profiles—in identifying individuals at an increased risk of fibrosis in the Mexican population.

Importantly, the superior performance of our composite fibrosis risk score compared with individual predictors highlights the value of integrating multiple metabolic and biochemical markers into a single tool. Risk prediction models play a critical role in clinical decision-making and resource allocation, especially in resource-limited settings, where efficient identification of individuals at higher risk is essential for targeted prevention and referral strategies.

Globally, fibrosis scores have shown variable performance across populations. For example, the AST/ALT ratio score was previously validated in a Latino population, showing a diagnostic accuracy of 67% (34). In contrast, in our sample, this index showed null discriminatory capacity, with AUROC values of 48.2 and 47.8%. This discrepancy may be explained by the fact that liver enzyme elevations are not consistently present in individuals with fibrosis (35), limiting the utility of AST/ALT as a standalone marker. Notably, when evaluated within multivariable models, both AST and the AST/ALT ratio were significantly associated with fibrosis status, underscoring the importance of contextualizing these biomarkers alongside other metabolic factors.

Similarly, previous evaluations of the BARD score have yielded mixed results. While some studies have reported good diagnostic accuracy (31, 32, 36, 37), others—including studies conducted in Latino populations—have found more modest performance, with AUROC values ranging from 65 to 70% (34, 38). Our findings are consistent with this latter body of evidence. Although simple variables such as obesity and T2D may help identify individuals at an increased risk of fibrosis, they appear insufficient for accurate classification in the Mexican population when used in isolation. The reduced performance of the BARD score may reflect genetic and ethnic heterogeneity, differences in the prevalence and clustering of metabolic risk factors, and variation in clinical and epidemiological contexts across populations.

Several limitations of this study should be acknowledged. First, the cohort comprises adults from a specific geographic area and occupational background, which may limit the representativeness of the broader Mexican adult population. Nonetheless, participants may reasonably reflect middle- and lower-income urban adults in central Mexico. Second, the cross-sectional design precludes the assessment of fibrosis progression over time. Third, missing laboratory data for some participants limited the evaluation of certain fibrosis indices, such as those requiring platelet counts. In addition, liver biopsy data were not available to directly compare the performance of the proposed score against histological assessment. However, prior studies have demonstrated that transient elastography provides diagnostic accuracy comparable to liver biopsy for fibrosis staging (24, 25, 39). It is important to note that the underlying etiology of liver disease in our cohort is predominantly metabolic-associated steatotic liver disease (MASLD). Individuals with a history of viral hepatitis were excluded, and alcohol consumption was very low in this population; only one male participant exceeded the threshold of two standard drinks per day and was excluded from the analysis. Therefore, our fibrosis score primarily reflects MASLD-related fibrosis rather than other causes of liver disease, which should be considered when interpreting and validating the model in other populations.

Despite these limitations, our study has notable strengths. The fibrosis risk score is based on laboratory and clinical measurements routinely available in primary care settings, enhancing its potential applicability. Moreover, the use of a well-characterized cohort allowed for robust internal validation, and the five-fold cross-validation analyses demonstrated stable out-of-sample performance.

The Mexican fibrosis risk score developed in this study accurately predicts fibrosis status using readily available anthropometric, biochemical, and clinical variables in Mexican adults. In contrast to previously developed scores, which showed limited predictive capacity in this population, our model demonstrated superior discriminatory performance. Future research should focus on external validation in independent Mexican cohorts and explore whether the inclusion of additional biomarkers or imaging modalities could further improve diagnostic accuracy.

## Conclusion

In this study, we developed and internally validated a simple, non-invasive fibrosis risk score based on routinely available clinical, anthropometric, and biochemical variables in a Mexican adult population. The proposed score demonstrated good diagnostic accuracy for identifying fibrosis status assessed by transient elastography (FibroScan), outperforming commonly used non-invasive tools, such as the BARD score and the AST/ALT ratio, in this population.

By relying on measurements that are widely accessible in primary care settings, this fibrosis risk score has the potential to facilitate early identification of individuals at an increased risk of fibrosis, support clinical decision-making, and reduce reliance on invasive diagnostic procedures. Although the robustness of the findings is supported by internal cross-validation and comprehensive statistical modeling, external validation in independent and more diverse populations is required. Future studies should also assess the clinical utility of this tool and explore whether the inclusion of additional biomarkers or complementary imaging modalities can further enhance its predictive performance.

## Data Availability

The raw data supporting the conclusions of this article will be made available by the authors, without undue reservation.

## Glossary

T2D: type 2 diabetes
MAFLD: metabolic-associated fatty liver disease
NASH: non-alcoholic steatohepatitis
NAFLD: non-alcoholic fatty liver disease
BMI: body mass index
AST: aspartate aminotransferase
ALT: alanine aminotransferase
AAR: AST/ALT ratio
APRI: AST-to-platelet ratio index
HWCS: Health Workers Cohort Study
IMSS: Instituto Mexicano del Seguro Social
GGT: gamma-glutamyl transferase
METAVIR: Meta-analysis of Histological Data in Viral Hepatitis
TG: triglycerides
HDL-c: high-density lipoprotein cholesterol
WC: waist circumference
SD: standard deviation
AUROC: area under the receiver operating characteristic
SBP: systolic blood pressure
DBP: diastolic blood pressure
AUC: area under the curve
95% CI: 95% confidence interval
