# Supplementary material for: Risk score to predict fibrosis among Mexican adults: results of the Health Workers Cohort Study
Source: Front Med (Lausanne). 2026 Mar 10;13:1699322. doi: 10.3389/fmed.2026.1699322 (PMC13008980; doi:10.3389/fmed.2026.1699322)
Supplement: Supplementary file 1 [file Data_Sheet_1.pdf]

Appendix 1

Risk score to predict fibrosis among Mexican adults: results of the Health Workers Cohort Study

Contents

Bayesian Probit Model Specification.. 2

Table S1. Description of predictors of fibrosis 2

Bayesian Probit Model Estimation 3

Table S2. Posterior means and 95% Credible intervals for probit coefficients. 3

Bayesian Probit Model Specification

We specified a Bayesian probit regression model. The dependent variable was a binary indicator of a fibrosis elastography result ($Y_i = 1$ with fibrosis; $Y_i = 0$ without fibrosis). A detailed description of all predictors included in the model is provided in Table S1.

The outcome variable was assumed to follow a Bernoulli distribution:

$$Y_i \sim Bernoulli(\pi_i) \qquad i = 1, .., n$$

$$\text{probit}(\pi_i) = \Phi^{-1}(\pi_i) = \beta_0 + \beta_1 x_{1i} + \cdots + \beta_k x_{ki}$$

For predictors which are known to be risk factors, we specified diffused half-normal prior distributions with a location parameter of zero and a variance of 1000 (i.e. a precision parameter of $\tau = 0.001$):

$\beta_j \sim HalfNormal(\mu = 0, \sigma^2 = 1000)$ for $j = 2, .., k$

For the intercept term and the probit coefficient corresponding to the female indicator variable, we specified normal prior distributions with mean zero and variance 1000, reflecting the absence of strong prior assumptions regarding the direction of their effects:

$\beta_j \sim Normal(\mu = 0, \sigma^2 = 1000)$ for $j = 0,1$

Table S1. Description of predictors of fibrosis

**Variable**	**Description**
x_1	Female indicator variable (=1 if female; =0 if male).
x_2	High triglycerides (=1 if triglycerides ≥150 mg/dL; =0 otherwise)
x_3	Insulin, standardized
x_4	GGT, standardized
x_5	Hypertension (=1 if SBP >135 mmHg or DBP >85 mmHg; =0 otherwise)
x_6	AST, standardized
x_7	High glucose (=1 if glucose ≥100 mg/dL; =0 otherwise)
x_8	Low HDL-c (=1 if HDL-c<40 in males or HDL-c <50 in females; =0 otherwise)
x_9	AST/ALT
x_{10}	Abdominal obesity (=1 if WC ≥102 in males or WC ≥88 in females; =0 otherwise)

SBP: Systolic blood pressure; DBP: Diastolic blood pressure; HDL-c: High density lipoprotein cholesterol; WC: Waist circumference; GGT: Gamma-glutamyl transferase; AST: Aspartate aminotransferase; ALT: Alanine aminotransferase.

Bayesian Probit Model Estimation

Bayesian probit regression coefficients were estimated using four Markov chain Monte Carlo (MCMC) chains, each run for 9,000 iterations, with the first 1,000 iterations discarded as burn-in and a thinning interval of 2. Posterior means of the probit coefficients, along with percentile-based 95% credible intervals, are presented in Table S2. A graphical summary of these results is provided in the main manuscript (Figure 3).

Convergence diagnostics indicated satisfactory model performance. All R-hat statistics for predictor coefficients were below 1.01, suggesting adequate convergence and good mixing of the MCMC chains. Only the intercept term in the subsample analysis exhibited an R-hat value marginally above 1.01.

Table S2. Posterior means and 95% Credible intervals for probit coefficients

Predictor	Subsample n=224		Full analysis sample n=295	
	**Posterior mean (95% CI)**	**R-hat**	**Posterior mean (95% CI)**	**R-hat**
Model constant	-3.150 (-4.297, -2.085)	1.00712	-2.789 (-3.703, -1.980)	1.01125
Female	-0.679 (-1.312, -0.055)	1.00051	-0.491 (-1.019, 0.042)	1.00224
High triglycerides	0.143 (0.005, 0.442)	1.00021	0.116 (0.004, 0.360)	1.00007
Insulin, standardized	0.168 (0.014, 0.382)	1.00000		
GGT, standardized	0.202 (0.015, 0.450)	1.00009	0.169 (0.022, 0.336)	1.00044
Hypertension	0.209 (0.008, 0.588)	1.00000	0.217 (0.011, 0.564)	1.00010
AST, standardized	0.433 (0.201, 0.669)	1.00045	0.401 (0.219, 0.585)	1.00057
High glucose	0.480 (0.053, 1.002)	1.00059	0.473 (0.076, 0.915)	1.00173
Low HDL	0.758 (0.250, 1.300)	1.00107	0.599 (0.179, 1.038)	1.00221
AST/ALT	0.871 (0.170, 1.607)	1.00460	0.660 (0.106, 1.295)	1.00903
Abdominal obesity	0.937 (0.267, 1.684)	1.00258	0.816 (0.287, 1.391)	1.00222

High triglycerides defied as triglycerides ≥150 mg/dL, High glucose defined as glucose ≥100 mg/dL, hypertension defined as systolic blood pressure >135 mmHg or diastolic blood pressure >85 mmHg, low high density lipoprotein cholesterol (HDL-c) defined as HDL-c <40 if male or HDL-c <50 if female, abdominal obesity defined as waist circumference ≥102 if male and ≥88 if female.
